# NINJ1: Bridging lytic cell death and inflammation therapy

**DOI:** 10.1038/s41419-024-07203-6

**Published:** 2024-11-15

**Authors:** Jinze Shen, Ruixiu Chen, Shiwei Duan

**Affiliations:** Key Laboratory of Novel Targets and Drug Study for Neural Repair of Zhejiang Province, School of Medicine, Hangzhou City University, Hangzhou, Zhejiang 310015 China

**Keywords:** Cell death, Cell signalling

## Abstract

NINJ1, a critical transmembrane protein in inflammation, governs diverse biological processes. Recent breakthroughs revealed NINJ1’s structural basis for plasma membrane rupture, which is directly linked to lytic cell death. This discussion explores NINJ1’s functions, focusing on its pivotal role in lytic cell death regulation and the latest advancements in targeted therapeutic interventions.

## NINJ1: A membrane protein implicated in inflammation

Lytic cell death, encompassing processes like pyroptosis, necrosis, and ferroptosis, is characterized by cell swelling and plasma membrane rupture (PMR) [1, 2]. PMR-induced release of intracellular proteins, including lactate dehydrogenase (LDH) and damage-associated molecular patterns (DAMPs), represents the final catastrophic event in cell death. PMR acts as a double-edged sword: it triggers the inflammatory response necessary for pathogen clearance but also contributes to tissue damage and chronic inflammation [1–3]. This chronic inflammation is associated with the development of various diseases, including diabetes, cardiovascular disease, and cancer [1].

In the exploration of lytic cell death, Ninjurin1 (NINJ1) has emerged as a key factor. Since its discovery in 1996, the implications of NINJ1 have extended far beyond its initial association with damaged nerve endings [4]. Its role in regulating inflammatory responses has gained increasing recognition [5]. Epidemiological studies have linked elevated NINJ1 expression to various inflammatory diseases, including nerve damage disorders [6] and vascular conditions like thoracic aortic dissection [7]. These conditions share a commonality: the presence of an inflammatory response, marked by cell death within the lesion, release of cellular contents, and subsequent repair processes. Elevated NINJ1 expression in damaged tissue regions suggests its pivotal role in the onset and progression of these inflammatory ailments, thereby highlighting its potential as a therapeutic target [7].

**Fig. 1:**
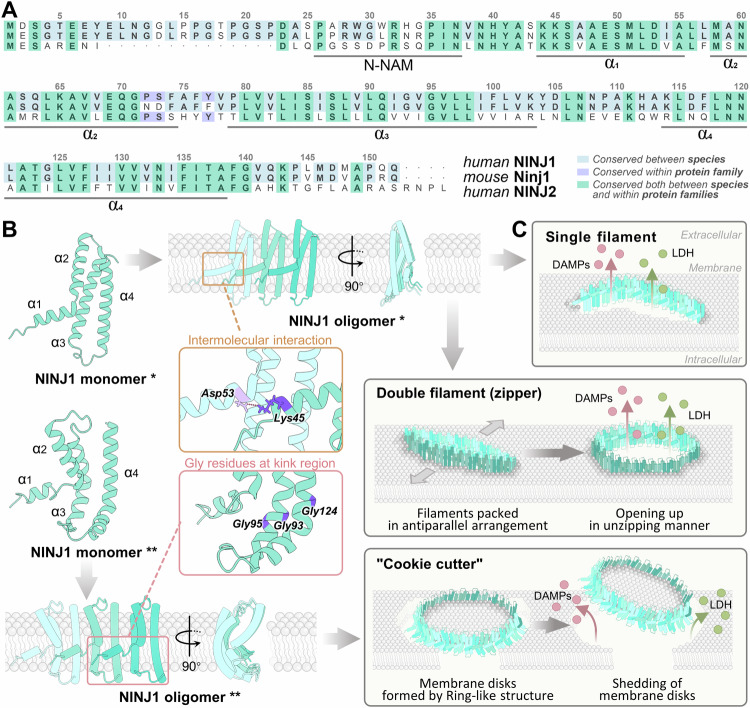
Structural basis of NINJ1-mediated PMR. **A** Primary Structure of NINJ1. The protein sequence of human NINJ1 is compared with mouse Ninj1 and human NINJ2. NINJ1 is highly conserved across species, with several key residues important for NINJ1-mediated plasma membrane repair (PMR)—specifically Lys45, Asp53, Gly93, Gly95, and Gly124—shared between human NINJ1 and mouse Ninj1. **B** Cryo-EM Structure of NINJ1. NINJ1 forms chain-like oligomers in a hand-in-hand configuration. Intermolecular interactions, such as the salt bridge between Lys45 on α1 and Asp53 on the adjacent α1, along with Gly residues at kink regions in the transmembrane (TM) helices, are crucial for NINJ1 oligomerization. The NINJ1 oligomer is amphipathic, with hydrophobic TM helices (α3 and α4) and hydrophilic α1 and α2. *Indicates the straight structure of NINJ1 reported by Degen M et al. (PDB: 8CQR), and ** indicates the curved structure reported by David L et al. (PDB: 8UIP). **C** Possible Models of NINJ1 Polymer. NINJ1 further aggregates to form polymers that trigger PMR. Three models currently explain how NINJ1 forms openings in the plasma membrane: the single filament and double filament (zipper) models proposed by Degen M et al., and the “cookie cutter” model proposed by David L et al.

The functional attributes of NINJ1 are closely intertwined with its structural characteristics. Serving as a highly conserved secondary transmembrane protein, NINJ1 boasts a distinctive structural configuration (Fig. 1). In the case of NINJ1, it consists of two hydrophobic transmembrane helices that together form the transmembrane (TM) domain, and also has an extracellular N-terminal domain, an extracellular C-terminal domain, and a cytoplasmic domain [5]. Notably, the structure of NINJ1 encompasses four α-helices, α1 to α4, which are arranged sequentially from the N- to C-terminus. Among these, α1 and α2 represent two amphipathic helices, roughly perpendicular to each other, while α3 and α4 constitute the TM helices [8]. Contrasting with the straight oligomeric structure of NINJ1 reported by Degen M et al. [8], the recent findings by David L et al. depict a curved structure for α3 and α4, featuring glycine residues at precise kink regions: Gly93 and Gly95 in α3 and Gly124 in α4 [3]. The unique structure of NINJ1 enables it to play a significant role in inflammatory responses and cell death [1, 3, 6]. Detailed exploration of the structure of NINJ1 provides a scientific foundation for understanding its regulatory mechanisms in cell biology.

## NINJ1-mediated plasma membrane rupture: unraveling the active dynamics of inflammation

Historically, PMR was considered a non-specific byproduct of cell death, but recent research has revealed a more complex picture. The pore size of the pyroptosis core pore-forming protein GSDMD is insufficient to release large molecules like LDH and DAMPs [9, 10]. This suggests an actively regulated mechanism is responsible for the release of macromolecular inflammatory contents after cell death. In 2021, Kayagaki N et al. demonstrated that PMR is not a passive osmotic lysis event but an actively regulated post-cell death lysis event. Their work identified NINJ1 as a crucial player in PMR, showing that oligomerized NINJ1 mediates the release of LDH and DAMPs, challenging prior assumptions about PMR [1, 9]. Furthermore, their research demonstrated that regardless of the cell death inducer used (pyroptosis, necrosis, or apoptosis), NINJ1 knockout could mitigate PMR to varying degrees without inhibiting cell death [1]. This suggests that NINJ1’s primary function lies not in cell death per se, but in the inflammatory activation response following cell death, offering a fresh perspective on the interplay between cell death and inflammatory reactions. Moreover, NINJ1 deficiency reduces the release of large molecules via PMR while leaving the GSDMD-dependent release of small molecules unaffected [1].

In its inactive state, NINJ1’s α1 and α2 helices do not directly interact with the outer cell membrane [3, 8, 9]. However, upon receiving death signals, such as inflammasome activation, NINJ1 monomers rapidly oligomerize through specific interactions. This process involves precise pairing and structural reorganization between NINJ1 molecules. According to the research conducted by Degen M et al., the oligomerization of NINJ1 commences with conformational alterations. The extracellular α helices (α1 and α2) insert into the membrane, thereby bridging adjacent monomers. Lys45 on α1 forms a salt bridge with Asp53 on an adjacent α1. Moreover, α3 and α4 interact through cation-π interactions, potentially involving Lys65 and Phe135 [8]. Additionally, α2 and α3 participate in intramolecular interactions via their hydrophobic patches [8]. David L et al. further noted that kink regions in the TM helices, Gly93 and Gly95 in α3 and Gly124 in α4, are essential for NINJ1’s function. Mutations in these residues (*e.g*., G95L and G124L) significantly inhibit cell lysis and LDH release [3, 8]. These structural interactions allow NINJ1 monomers to oligomerize on the cell membrane, forming a continuous chain-like structure (Fig. 1). The α1 helix acts as an “arm,” linking individual NINJ1 monomers, while the amphipathic nature of the chain oligomers stabilizes their anchoring on the membrane. The hydrophobic TM helix faces the membrane, and the hydrophilic α1 and α2 helices create a driving force that destabilizes and damages the membrane [3]. While computational models like AlphaFold have advanced our understanding of protein structures, they may not fully capture dynamic protein interactions in biological environments. Therefore, experimental techniques, including cryo-electron microscopy, are essential for elucidating the precise structure and function of proteins such as NINJ1 during cell death [3, 9].

Several models have been proposed to explain how NINJ1 forms openings in the cell membrane, including the single filament, double filament (zipper), and “cookie cutter” models (Fig. 1). The single filament model, proposed by Degen M et al., posits that NINJ1 monomers form a polymer with a hydrophilic side that repels the lipid membrane, resulting in membrane instability and pore formation [8]. The double filament model suggests that two single filaments stack together in an antiparallel manner. The outer side of the structure is hydrophobic and connected to the lipid membrane, while the inner side of the two filaments is a hydrophilic structure. In this model, the dual-filament structure opens on both sides of the filament, releasing the contents from the cavities formed on the inner sides of the two filaments, akin to the opening mechanism of a zipper. This “unzipping” may create larger holes in the membrane, promoting PMR [8]. David L et al. introduced the “cookie cutter” model, in which NINJ1 oligomers form ring-like structures. The inner surface of the ring is tightly bound to the cell membrane through hydrophobic interactions, while the hydrophilic structure on the outer surface of the ring pushes the cell membrane open, leading to the formation and shedding of NINJ1-encircled membrane disks. This unique “cookie cutter” mechanism enables NINJ1 to punch multiple holes in the cell membrane [3]. As these pores accumulate beyond the cell’s capacity to repair them, PMR is triggered, resulting in the release of LDH and DAMPs—a mechanism distinct from GSDMD pores [3].

## Activation and regulation of NINJ1: mechanism of an inflammatory membrane protein under multiple triggering factors

**Fig. 2:**
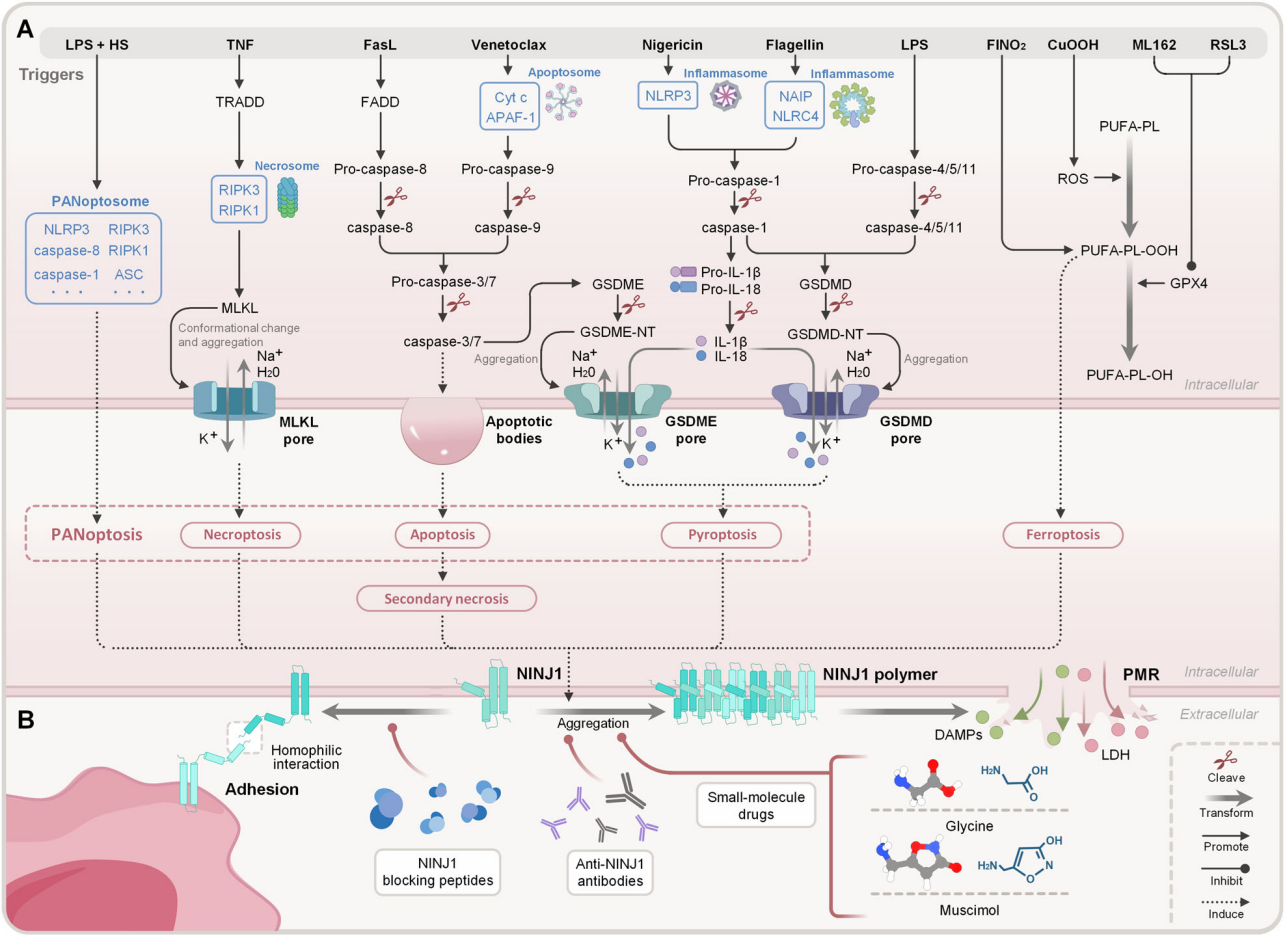
Role of NINJ1 in various types of lytic cell death and targeted therapeutic strategies. **A** Cell death signals and NINJ1 activation. NINJ1 mediates PMR in response to various cell death stimuli, but the factors that directly trigger NINJ1 activation remain to be determined. Knocking out NINJ1 results in varying degrees of inhibition of PMR across different types of cell death. In most cases, NINJ1 facilitates PMR without affecting overall cell death, such as in pyroptosis. **B** NINJ1 targeted therapy strategy. Prior to establishing the link between NINJ1 and PMR, researchers used NINJ1 blocking peptides to competitively inhibit NINJ1’s N-NAM, disrupting its homophilic binding and reducing immune cell adhesion and infiltration. Following the identification of NINJ1’s role in PMR, monoclonal antibodies were developed to inhibit NINJ1-mediated PMR; for instance, Clone D1 binds to the extracellular structure of NINJ1, preventing its aggregation. Additionally, small-molecule drugs like glycine and muscimol are being investigated for their ability to disrupt NINJ1 accumulation on the plasma membrane, effectively suppressing PMR. CuOOH cumene hydroperoxide, DAMPs damage associated molecular patterns, LDH lactate dehydrogenase, LPS lipopolysaccharide, PMR plasma membrane rupture, PUFA-PL polyunsaturated fatty acid-containing phospholipid, ROS reactive oxygen species RSL3 RAS-selective lethal 3 SLO streptolysin O, TNF tumor necrosis factor.

Nevertheless, what triggers the oligomerization of NINJ1? As shown in Table 1 and Fig. 2, current research primarily focuses on artificially inducing various types of cell death to activate NINJ1, investigating its role in PMR and lytic cell death by manipulating NINJ1 expression.

**Table 1:** The function of NINJ1 in various types of cell death.

Types of cell death	Triggers	Effects of specific gene knockout	Effects of NINJ1 knockout in vitro and vivo	Ref.
Pyroptosis	LPS	GSDMD^-/-^: PMR (↓↓↓) and IL-1β (↓↓↓).	PMR ( ↓ ↓ ↓ ), IL-1β(ns), and cell death (ns) in vitro (BMDMs cell line); Anti-bacterial host defense (↓) in vivo (acute septic shock model based on C57BL/6 N mice infected with *Citrobacter rodentium*).	[1]
		—	PMR (↓↓↓) in vitro (BMDMs cell line); *NINJ1 monoclonal antibody (clone D1) can inhibit PMR in vitro (BMDMs cell line).	[5]
		GSDMD^-/-^: PMR (↓↓↓) and IL-1β (↓↓↓)	PMR (↓↓↓) and cell death (ns) in vitro (BMDMs cell line).	[8]
		—	*Muscimol can inhibit the polymerization of NINJ1 and PMR in vitro (BMDMs cell line) inhibit serum LDH and BUN, promote survival in vivo (acute septic shock model based on C57BL/6 J mice infected with *Salmonella*).	[15]
		—	PMR (↓↓↓) in vitro (iBMDMs and hMDMs cell lines). *Glycine can inhibit the polymerization of NINJ1 and PMR in vitro (iBMDMs and hMDMs cell lines).	[14]
	Nigericin	GSDMD^-/-^: PMR (↓↓) and IL-1β (↓↓↓).	PMR ( ↓ ↓ ), IL-1β(ns), and cell death (ns) in vitro (BMDMs cell line). *NINJ1 monoclonal antibody can inhibit PMR in vitro (BMDMs cell line).	[1]
		—	PMR (↓↓↓) and cell death (ns) in vitro (BMDMs cell line); *NINJ1 monoclonal antibody (clone D1) can inhibit the polymerization of NINJ1 and PMR in vitro (BMDMs cell line).	[5]
		GSDMD^-/-^: PMR (↓↓↓) and IL-1β (↓↓↓)	PMR (↓↓↓) and cell death (ns) in vitro (HeLa and BMDMs cell lines).	[8]
		—	*Muscimol can inhibit PMR in vitro (BMDMs cell line).	[15]
		—	PMR (↓↓↓) in vitro (iBMDMs and hMDMs cell line). *Glycine can inhibit the polymerization of NINJ1 and PMR in vitro (iBMDMs and hMDMs cell line).	[14]
	*Salmonella typhimurium*	GSDMD^-/-^: PMR (↓↓↓) and IL-1β (↓↓↓)	PMR (↓↓↓) and cell death (ns) in vitro (BMDMs cell line).	[8]
	Poly(dA:dT)	GSDMD^-/-^: PMR (↓↓↓) and IL-1β (↓↓↓)	PMR (↓↓↓) and cell death (ns) in vitro (BMDMs cell line).	[8]
	Anthrax lethal toxin	—	*Muscimol can inhibit PMR in vitro (BMDMs cell line)	[15]
	Flagellin	GSDMD^-/-^: PMR (↓↓↓).	PMR ( ↓ ↓ ↓ ), GSDMD (ns), and cell death (ns) in vitro (BMDMs cell line).	[1]
		—	PMR (↓↓↓) and cell death (ns) in vitro (BMDMs cell line); *NINJ1 monoclonal antibody (clone D1) can inhibit PMR in vitro (BMDMs cell line).	[5]
Apoptosis	Venetoclax	GSDMD^-/-^: PMR (ns); GSDME^-/-^: PMR (ns).	PMR (↓↓) and cell death (ns) in vitro (BMDMs cell line).	[1]
		—	PMR (↓↓) in vitro (iBMDMs cell line). *Glycine can inhibit PMR in vitro (iBMDMs cell line).	[14]
		—	PMR (↓↓↓) in vitro (BMDMs cell line); *NINJ1 monoclonal antibody (clone D1) can inhibit PMR in vitro (BMDMs cell line)	[5]
	Doxorubicin	GSDMD^-/-^: PMR (ns).	PMR (↓) in vitro (BMDMs cell line).	[1]
		—	PMR (↓↓) and cell death (ns) in vitro (BMDMs cell line). *NINJ1 monoclonal antibody (clone D1) can inhibit PMR in vitro (BMDMs cell line).	[5]
	Cisplatin	GSDMD^-/-^: PMR (ns).	PMR (↓↓) in vitro (BMDMs cell line).	[1]
	FasL	GSDMD^-/-^: PMR (ns).	PMR (↓) in vitro (BMDMs cell line).	
	TNF + actinomycin D	—	PMR (↓↓) in vitro (BMDMs cell line); Serum LDH, ALT, AST, IL-18, and HMGB1 (↓↓↓) in vivo (fulminant hepatitis model based on C57BL/6 N mice induced with TNF plus D-galactosamine); *NINJ1 monoclonal antibody (clone D1) can inhibit PMR in vitro (BMDMs cell line) and serum LDH, ALT, AST, IL-18, and HMGB1 in vivo (fulminant hepatitis model based on C57BL/6 N mice induced with TNF plus D-galactosamine).	[5]
	TNF + AZD 5582	GSDMD^-/-^: PMR (ns).	PMR (↓↓) and cell death (ns) in vitro (BMDMs cell line).	[8]
	Staurosporine	—	*Muscimol can inhibit PMR in vitro (BMDMs cell line)	[15]
Secondary necrosis	Etoposide	MLKL^-/-^: PMR (ns); GSDME^-/-^: PMR (↓↓↓); GSDME^-/-^ NINJ1^-/-^: PMR (↓↓↓).	PMR (↓↓↓), and cell death (ns) in vitro (MEFs and RAW264.7 cell lines).	[13]
Parthanatos	H_2_O_2_	—	PMR (↓↓), cell death (ns) in vitro (MEFs and RAW264.7 cell lines).	[13]
	MNNG	—	PMR (↓↓), cell death (ns) in vitro (MEFs cell line).	
Ferroptosis	ML162	GSDME^-/-^: PMR (↓); NLRP3^-/-^: PMR (ns); GSDMD^-/-^: PMR (ns); RIPK3^-/-^ CASP-8^-/-^: PMR (ns); MLKL^-/-^: PMR (ns).	PMR (↓↓) in vitro (BMDMs, RAW264.7, MEFs, and NIH/3T3 cell lines).	[2]
		—	PMR (↓↓↓) and cell death (ns) in vitro (MEFs and RAW264.7 cell lines).	[13]
	RSL3	NLRP3^-/-^: PMR (ns); GSDMD^-/-^: PMR (ns); GSDME^-/-^: PMR (ns); RIPK3^-/-^ CASP-8^-/-^: PMR (ns); MLKL^-/-^: PMR (ns).	PMR (↓↓) in vitro (BMDMs, RAW264.7, MEFs, and NIH/3T3 cell lines).	[2]
		—	PMR (↓↓) and cell death (ns) in vitro (MEFs and RAW264.7 cell lines).	[13]
	CuOOH	NLRP3^-/-^: PMR (ns); GSDMD^-/-^: PMR (ns); GSDME^-/-^: PMR (ns); RIPK3^-/-^ CASP-8^-/-^: PMR (ns); MLKL^-/-^: PMR (ns).	PMR (↓↓) and cell death (ns) in vitro (BMDMs, RAW264.7, MEFs, and NIH/3T3 cell lines).	[2]
	Erastin	—	PMR (ns) in vitro (MEFs and RAW264.7 cell lines).	[13]
	FINO2	—	PMR (ns) in vitro (MEFs cell line).	
Necrosis	LLO	GSDMD^-/-^: PMR (ns).	PMR (↓↓) in vitro (BMDMs cell line).	[1]
	Pneumolysin	—	PMR (↓↓) in vitro (iBMDMs cell lines). *Glycine can inhibit PMR in vitro (iBMDMs cell line).	[14]
	SLO	GSDMD^-/-^: PMR (ns).	PMR (↓↓) in vitro (BMDMs cell line).	[1]
Necroptosis	TNF + zVAD	MLKL^-/-^: PMR (↓↓↓).	PMR (↓) in vitro (BMDMs cell line).	[1]
		—	PMR (↓) in vitro (BMDMs cell line); *NINJ1 monoclonal antibody (clone D1) cannot inhibit PMR in vitro (BMDMs cell line).	[5]
	zVAD + BV6 + TNF	—	*Glycine cannot PMR in vitro (hMDMs cell line).	[14]
PANoptosis	LPS and heat stress	GSDMD^-/-^: PMR (ns); GSDME^-/-^: PMR (ns); MLKL^-/-^: PMR (ns); caspase-8^-/-^RIPK3^-/-^: PMR (↓↓↓).	PMR (↓↓), caspase-1 (ns), caspase-11 (ns), GSDMD (ns), GSDME (ns), and cell death (↓) in vitro (BMDMs cell line).	[12]
Cellular swelling	Oligomycin (ATP depletion)	GSDMD^-/-^: PMR (ns); GSDME^-/-^: PMR (ns).	PMR (↓↓) in vitro (BMDMs cell line).	[1]

”↓↓↓“ significant decline, “↓↓“ moderate decline, “↓“ slight decline, ns not significant.

*ALT* alanine aminotransferase, *AST* aspartate aminotransferase, *BUN* blood urea nitrogen, *CuOOH* cumene hydroperoxide, *HMGB1* high mobility group box 1, *LDH* lactate dehydrogenase, *LLO* listeriolysin O, *LPS* lipopolysaccharide, *MNNG* methylnitronitrosoguanidine, *PMR* plasma membrane rupture, *RSL3* RAS-selective lethal 3, *SLO* streptolysin O, *STS* staurosporine, *TNF* tumor necrosis factor.

Specific signals trigger NINJ1 activation in different types of lytic cell death, with certain proteins playing crucial roles [11]. For instance, GSDMD is essential in pyroptosis [1], MLKL in necroptosis [1], and caspase-8/RIPK3 in PANoptosis [12]. However, it’s important to note that the same activation signal can have varying roles across different lytic cell death modes. For example, GSDMD, a key activator of NINJ1 in pyroptosis, is not critical for NINJ1 activation in PANoptosis induced by lipopolysaccharide (LPS) plus heat stress. In this context, GSDMD knockout does not affect NINJ1 activation or PMR occurrence, even though IL-1β release confirms GSDMD activation under these conditions [12]. Furthermore, research by Dondelinger Y et al. revealed that NINJ1 oligomerization can also be triggered by cell swelling. They demonstrated that reducing the osmotic pressure of the culture medium can induce NINJ1 oligomerization without inducing cell death. To confirm this, they used poly(ethylene glycol) as an osmotic protectant and reported that PEG4000 slowed PMR caused by cell swelling, whereas PEG400, with a smaller hydrated radius, did not [13].

The role of NINJ1 varies across different lytic cell death processes triggered by different factors. In many forms of cell death, such as pyroptosis [1], apoptosis [1], ferroptosis [2], necrosis [14], and secondary necrosis [13], NINJ1 regulates PMR and the release of DAMPs without directly affecting cell death. However, Han JH et al. reported that in PANoptosis, NINJ1 not only mediates membrane rupture and DAMP release but also may influence the cell death process itself. Their results showed that under LPS and heat stress, CRISPR-mediated knockout of NINJ1 reduced the uptake of propidium iodide (PI) by BMDMs, though it did not inhibit the processing and release of IL-1β and IL-18 [12]. Similar phenomena were also observed in ferroptosis induced by cumene hydroperoxide (CuOOH), RSL3, or ML162. Although the absence of NINJ1 could not reduce the final percentage of PI-positive cells, it could effectively dampen the rising curve of the percentage of PI-positive cells, that is, delay the occurrence of cell death [2].

In certain types of lytic cell death, such as pyroptosis [1], NINJ1 holds an important role in PMR. Nevertheless, due to the limitations of the experiment’s scope [1, 8], it remains unclear whether NINJ1 is truly indispensable for PMR in response to all triggers of pyroptosis. In others, such as apoptosis [1], necrosis [1], necroptosis [1], parthanatos [13], and ferroptosis [2, 13], NINJ1 knockout only partially alleviates or has no impact on PMR. For instance, in necroptosis, MLKL knockout significantly reduces PMR, surpassing the effect of NINJ1 knockout, suggesting that MLKL may activate a PMR mechanism independent of NINJ1 [1].

As our understanding of NINJ1-mediated PMR deepens, a more profound comprehension of the molecular mechanism of glycine in stabilizing the plasma membrane has also emerged. Traditionally perceived as an osmoprotectant mitigating osmotic pressure-induced pyroptotic membrane rupture and preventing LDH and DAMPs release [9, 15], glycine’s protective role has been further elucidated. A study demonstrated its ability to inhibit PMR [14], safeguarding the plasma membrane integrity of macrophages stimulated by pyroptotic, necrotic, or apoptotic inducers—a protective effect akin to NINJ1 deletion [1, 14]. Moreover, in NINJ1 knockout cells, glycine failed to exhibit additional protective effects on the cell membrane. This study also revealed the ability of glycine to reduce NINJ1 aggregation in the plasma membrane. Consequently, the protective function of glycine on the plasma membrane likely stems from its interference with NINJ1 aggregation, although the precise mechanism awaits further experimental exploration. This perspective elucidates a previous study by Xia S et al., explaining glycine’s ability to protect cells from PMR and selectively inhibit LDH and DAMPs release without affecting IL-1β release [9, 14]. However, the molecular mechanism by which glycine affects NINJ1 aggregation on the cell membrane remains unclear, partly due to the incomplete understanding of NINJ1 activation itself.

In conclusion, the specific factor that directly activates the oligomerization of NINJ1 remains unidentified. The role of NINJ1 in different types of lytic cell death is intricate and not fully understood. The influence of the intracellular and extracellular environments on it, as well as potential interactions with other molecules, are areas that require further exploration. Additional proteins may also regulate PMR in various types of lytic cell death, highlighting the need for further research.

## NINJ1-targeted therapies: unraveling new horizons in inflammatory disease management

Before its link to lytic cell death was discovered, NINJ1 was known to mediate homophilic binding through its N-NAM domain, playing a key role in immune cell adhesion to the vascular endothelium—an important aspect of the immune response [16]. Based on this, researchers have designed NINJ1-blocking peptides like NINJ1_26-37_ and NINJ1_16-45_ to intervene in related immune processes.

This disruptive potential was first conceptualized in 2012, demonstrating significant efficacy in hindering immune cell infiltration into the central nervous system, offering promise for the treatment of diseases like multiple sclerosis in preclinical models [17]. Further underlining the potency of NINJ1_26-37_, studies have validated its ability to inhibit key inflammatory factors induced by LPS stimulation. It curtails the expression of ICAM-1, MCP-1, and IL-6 in endothelial cells, while reducing TNF-α and IL-6 levels in macrophages. Encouraging outcomes were observed in mouse models of polymicrobial peritonitis, where NINJ1_26-37_ administration significantly decreased plasma levels of TNF-α, IL-6, and IL-10, mitigating systemic inflammation, liver damage, and pulmonary endothelial dysfunction, ultimately increasing overall survival [18].

As the involvement of NINJ1 in lytic cell death has become clearer, DAMPs released during NINJ1-dependent PMR have been identified as key drivers of inflammatory responses following cell death. These DAMPs exacerbate inflammation and can cause prolonged tissue and organ damage in chronic conditions [19]. Inhibiting NINJ1 activity to reduce DAMP release offers a promising approach for controlling inflammation and minimizing tissue damage. As shown in Fig. 2, initial progress has been made in developing antibody therapies and small-molecule drugs targeting NINJ1, showing anti-inflammatory potential.

Notably, NINJ1-antibody therapy, exemplified by clone D1, a mouse NINJ1-specific IgG2a monoclonal antibody, exhibited exceptional promise. Clone D1 selectively targets the C-terminal epitope of mouse NINJ1, effectively impeding NINJ1 oligomerization on the membrane [5]. In mouse bone marrow-derived macrophages, clone D1 demonstrates a dose-dependent inhibition of NINJ1-dependent PMR, without impeding the formation of bubble-like membrane herniations during nigericin, intracellular lipopolysaccharide, or flagellin-induced pyroptosis [5]. Additionally, clone D1 significantly inhibits PMR during apoptosis induced by doxorubicin, venetoclax, or TNF plus actinomycin. However, it does little to alleviation of PMR during necroptosis induced by TNF plus the pan-caspase inhibitor zVAD, mirroring the slight protective effect observed with NINJ1 deletion [1, 5]. However, whether induced by pyroptosis, apoptosis, or necrosis, clone D1 cannot prevent the ultimate outcome of cell death [5]. In mouse hepatitis models, clone D1 exhibits significant reductions in PMR and the release of pro-inflammatory DAMPs, showcasing its potent efficacy [5]. While antibody-targeted therapies have demonstrated significant potential, they also encounter certain challenges. For instance, monoclonal antibodies derived from mice may provoke human anti-mouse antibody reactions, restricting their clinical utility. To improve these therapies, humanization techniques can be used to fuse the Fab region of mouse monoclonal antibodies with the human Fc region, reducing immunogenicity and enhancing clinical safety. However, novel antibody therapy like NINJ1 monoclonal antibodies necessitate extensive clinical trials and long-term data to establish their safety and efficacy in disease management. Moreover, antibody treatments are often expensive, rendering them financially inaccessible to many patients. Hence, addressing these obstacles and enhancing the accessibility and affordability of antibody treatment have emerged as pressing issues requiring resolution.

In addition to the development of antibodies, research into small-molecule drugs targeting NINJ1-mediated PMR is also progressing. A drug widely studied as an agonist for neuronal GABA_A_ receptors, muscimol, whose structure is similar to glycine, exerts a cell membrane protective effect that cannot be replaced or antagonized by other GABA_A_ receptor agonists [15]. Similar to glycine, muscimol can shield cells from PMR following pyroptosis stimulation by inhibiting the oligomerization of NINJ1 on the cell membrane. However, it does not influence the formation of GSDMD pores or the release of IL-1β [14, 15]. In the LPS-induced sepsis model, muscimol demonstrated protective effects against septic shock and secondary renal failure [15]. However, it’s essential to note that once muscimol is removed from the cellular environment, previously protected cells resume undergoing lysis. This indicates that the protective effect of muscimol relies on its continuous presence. Once removed, the protective effect dissipates, allowing cell lysis to resume [15]. This differs from the mechanisms of action of other antipyroptotic small molecule drugs, such as dimethyl fumarate (DMF) and disulfiram (DSF). These drugs, which inhibit pyroptosis by forming covalent bonds with GSDMD cysteine residues [20, 21], present a challenge when applied to NINJ1, as it lacks cysteine residues. Consequently, alternative drug design strategies focusing on other amino acid residues, such as lysine-directed covalent ligands, are being explored [22]. As previously mentioned, Lys45 in the α1 helix of NINJ1 forms a salt bridge with Asp53 in an adjacent NINJ1 molecule through electrostatic interactions, likely playing a key role in NINJ1 oligomerization [8]. Targeting lysine residues on NINJ1, especially Lys45, presents a promising strategy for drug design. However, since the ε-amino group of lysine exhibits low nucleophilicity under physiological conditions, researchers must account for this when designing electrophiles. Enhancing the electrophile’s reactivity toward lysine would be essential for effectively forming covalent bonds. Similarly, the β-carboxyl group of aspartic acid is another potential target for NINJ1 drug development, though its weak electrophilicity and dependence on the surrounding chemical environment must be carefully considered [23]. Researchers must analyze these environmental factors to design more precise and effective drugs.

When delving into the potential and challenges of NINJ1-targeted therapy, we must acknowledge the contributions of Kayagaki N et al. [5] and den Hartigh AB et al. [15]. Arguably the study by Kayagaki N et al. is the first to even demonstrate that PMR is therapeutically targetable in vivo. Likewise, the study by den Hartigh AB et al. opens up the possibility of small molecule intervention in NINJ1-mediated PMR. When anti-pyroptosis treatments (*e.g*., GSDMD inhibitors) are compared with NINJ1-targeted therapy, we can discern unique advantages of the latter. NINJ1 targeted therapy can address PMR after various types of lytic cell death, not limited to pyroptosis. However, treatments for NINJ1-mediated PMR also share a common limitation: specifically inhibiting NINJ1 primarily affects the inflammatory response after cell death, rather than the cell death process itself.

Overall, the feasibility of NINJ1-targeted therapy in inflammation is beginning to emerge, promising a future where inflammatory disorders can be effectively controlled. Although NINJ1 inhibition may not prevent cell death directly, it can significantly reduce the inflammatory response by curbing the release of DAMPs and inflammatory mediators. This presents NINJ1 as a promising target for treating inflammatory conditions [1, 19]. For instance, NINJ1 has been identified as a risk factor in conditions such as ischemic stroke [24] and type 2 diabetes [25]. While more research is needed, inhibiting NINJ1 could prove beneficial for patients suffering from persistent inflammation. But at present, whether it is monoclonal antibodies or small molecules, there is still a long way to go before they can be used in clinical practice. To attain widespread clinical application, overcoming a series of challenges—including side effect management, drug stability, and long-term effect evaluation—is imperative. Drawing on successful experiences and lessons from other anti-pyroptosis treatment strategies, along with its unique advantages, NINJ1-targeted therapy offers promise for breakthroughs in future research. This endeavor holds the potential to bring new hope to the control of inflammatory diseases and the recovery of patients.

## Concluding remarks

NINJ1, a multifunctional regulatory protein, holds a pivotal role in organismal health and disease progression. Particularly in lytic cell death, NINJ1 assumes a central position, facilitating cell membrane rupture through specific oligomer formation and subsequent release of DAMPs, thus serving as a crucial trigger for the inflammatory response. Recent studies have indicated that inhibiting pro-inflammatory effects of NINJ1 through monoclonal antibodies or small-molecule drugs could provide new strategies for treating infections and inflammatory diseases. This could allow damaged or infected cells to die without causing excessive inflammation, reducing disease burden and promoting tissue repair. However, there is a possibility that NINJ1-targeted therapies could impair pathogen clearance, as NINJ1-mediated DAMP release may help eliminate microorganisms in some infections [13]. Therefore, similar to glucocorticoid treatments, NINJ1-targeted therapies may need to be combined with causal treatments (*e.g*., antibiotics for infections) to ensure that inflammation is controlled while also addressing the underlying causes of excessive cell death.

Though not yet widely applied clinically, these treatment strategies hold promise for the future. Future studies should focus on evaluating the therapeutic potential of NINJ1, optimizing treatment doses and strategies, and continuously monitoring the effects and safety of these treatments. Thus, gaining a profound understanding of NINJ1’s molecular mechanism in lytic cell death, coupled with evolving NINJ1-targeted therapy, is crucial in establishing a translational pathway from basic to clinical medicine. This holds the promise of ushering in new hope in combating inflammation, paving the way for innovative treatments and breakthroughs in medical science.
